# Association of FSHR, LH, LHR, BMP15, GDF9, AMH, and AMHR polymorphisms with poor ovarian response in patients undergoing *in vitro* fertilization

**DOI:** 10.5935/1518-0557.20210004

**Published:** 2021

**Authors:** Arivaldo José Conceição Meireles, João Paolo Bilibio, Pânila Longhi Lorenzzoni, Emily De Conto, Fábio Costa do Nascimento, João Sabino da Cunha-Filho

**Affiliations:** 1 Clinica Pronatus Centro de Reprodução Humana, Belém, Pará, Brazil; 2 Programa de Pós Graduação de Ciências Médicas da Universidade Federal do Rio Grande do Sul, Porto Alegre, Rio Grande do Sul, Brazil; 3 Department of Obstetrics and Gynecology, Universidade Federal do Pará, Belém, Pará, Brazil; 4 Clínica Insemine Centro de Reprodução Humana, Porto Alegre, Brazil

**Keywords:** poor ovarian response, polymorphisms, ovarian reserve, IVF, SNPs

## Abstract

**Objective:**

This paper aimed to assess the correlation between LH, LHR, GDF9, FSHR, AMH, AMHR2, and BMP15 polymorphisms, which are related to follicular development, and decreased ovarian response in women undergoing controlled ovarian hyperstimulation (COH) for IVF.

**Methods:**

This age-matched case-control study included three or four controls per woman undergoing COH. Controls were women with normal ovarian response (NOR) and cases were women with poor ovarian response (POR) in oocyte retrieval (three or fewer oocytes). DNA was extracted from peripheral blood and potential associations with gene polymorphisms related to follicular development (LH, LHR, GDF9, FSHR, AMH, AMHR2, and BMP15) were analyzed.

**Results:**

Sixty-six patients were included, 52 in the NOR and 14 in the POR group. Two GDF9 polymorphisms were associated with follicular response after COH, one associated with POR - the presence of a mutant polymorphism in heterozygosis and homozygosis of the GDF9 398-39 (C to G) [23% NOR versus 68% POR (OR 4.01, CI 1.52-10.6, *p*=0.005)] - and another associated with protective response - the presence of normal homozygosis of GDF9 (C447T) [19.2% NOR versus 50% POR (OR 0.34, IC 0.14-0.84, *p*=0.019)]. No additional associations were found between the other analyzed polymorphisms and POR.

**Conclusions:**

This study found that GDF9 appears to play an important role in follicular development, whereas polymorphisms in its DNA chain may negatively affect ovarian reserve, such as 398-39 (C to G), or positively, as seen in C447T.

## INTRODUCTION

Approximately 10% of women seeking fertility treatment have diminished ovarian reserve (DOR), defined as a decreased number or quality oocytes (Ferraretti *et al*., 2011). Poor ovarian response (POR) is defined as meeting two or three of the following criteria: age greater than 39 years; prior POR to conventional stimulation protocols (less than three oocytes retrieved); and abnormal ovarian reserve testing (Ferraretti *et al*., 2011). DOR can have multiple etiologies, including autoimmunity, idiopathic, iatrogenic, and genetic causes, and single nucleotide polymorphisms (SNPs) (Goswami & Conway, 2005; Greene *et al*., 2014). The impact of mutations and polymorphic variant genes involved in folliculogenesis is still uncertain.

Polymorphisms exist within genes and are a source of variation between individuals. Gene association studies have identified a number of SNPs that affect gonadotropin, steroid, and transforming growth factor beta (TGFβ) pathways, which include TGF-β, anti-Müllerian hormone (AMH), activins, inhibins, bone morphogenetic proteins (BMPs), and growth differentiation factors (GDFs) involved in ovarian response (Greene *et al*., 2014). Most of them affect mRNA levels or the protein sequence, and thus lead to quantitative functional protein variations that may account for the observed inter-individual variability in controlled ovarian hyperstimulation (COH) (Levi *et al*., 2001; Greene *et al*., 2014).

Growth differentiation factor 9 (GDF9) and bone morphogenetic protein 15 (BMP-15), members of the TGFβ superfamily, are potent regulators of folliculogenesis and ovulation and are both expressed in oocytes from early stage follicles (Aaltonen *et al*., 1999; Chang *et al*., 2016; Sanfins *et al*., 2018). With respect to COH phenotypes, GDF9 and BMP15 alleles have been associated with stimulation outcome (Moron *et al*., 2006; Wang *et al*. 2010a; Hanevik *et al*., 2011; Bilibio *et al*., 2020). Several genetic variants of GDF9 have been identified, and their correlation with POR has been noted, suggesting that these variants contribute to aberrant follicular development and oocyte loss (Di Pasquale *et al*., 2004; Shimizu *et al*., 2004; Abir *et al*., 2008; Wang *et al*., 2010a;b). Other variant alleles of BMP15 were associated with increased follicle production in COH, such as the 9C>G polymorphism, which was associated with high response to ovarian stimulation (Moron *et al*., 2006; Hanevik *et al*., 2011).

The follicle stimulating hormone receptor (FSHR) carries more than two thousand SNPs, and one polymorphism, Asn608Ser, was found to have an important impact on ovarian response (Livshyts *et al*., 2009; Sheikhha *et al*., 2011; Pabalan *et al*., 2014). Homozygous mutant women with this polymorphism have higher baseline FSH levels, require higher FSH doses during COS, and present lower estradiol levels than heterozygous and normal homozygous women. Another study showed that women homozygous for the Ser680 variant had a greater number of mature oocytes than women homozygous for the Asn680 variant (Lawson *et al*., 2003; Ferrarini *et al*., 2013).

AMH (Ile49Ser) and AMHR polymorphisms (482A>G) have been associated with variations in estradiol levels and may modulate FSH sensitivity (de Boer *et al*., 2002). In regard to LH polymorphisms, variant 8Arg-15Thr in women undergoing IVF is more frequently seen among poor responders to rFSH and women with ovarian resistance to rFSH who, despite requiring higher rFSH dosages, have fewer oocytes retrieved (Alviggi *et al*., 2009).

This paper aimed to assess the correlation between LH, LHR, GDF9, FSHR, AMH, AMHR2, and BMP15 polymorphisms, which are related to follicular development, and decreased ovarian response in women undergoing COH for IVF.

## MATERIALS AND METHODS

This age-matched case-control study included three or four controls per case involving women undergoing COH for IVF. Controls were women with normal ovarian response (NOR) and cases were women with POR in oocyte retrieval (three or fewer oocytes). The study was carried out at the Pronatus Assisted Reproduction Center and at the Federal Univesity of Rio Grande do Sul. The study was approved by the National Ethics and Research Committee on Human Beings and granted certificate no. 25525413.0.0000.5327 by the Ethics Committee of the Hospital de Clínicas de Porto Alegre (an Institutional Review Board equivalent).

Sixty-six patients were included in the study. The inclusion criteria were as follows: age between 30 and 39 years; presence of both ovaries; diagnosis of infertility (more than one year) by male factor, tubal factor (confirmed by hysterosalpingography or videolaparoscopy), or unexplained causes; and no prior IVF treatment. Three or four controls were matched by age to each case. The exclusion criteria were as follows: endocrine disorders; polycystic ovary syndrome; prior chemotherapy; abnormal karyotype; and other factors affecting ovarian function, such as ovarian surgery and endometrioma.

The patients underwent antral follicle counts on Day 2 or 3 of the menstrual cycle and were tested for FSH, LH, estradiol, and anti-Müllerian hormone (AMH) levels. Ovarian stimulation was initiated on Day 3 of the menstrual cycle with recombinant FSH (Puregon, Organon) and included r-FSH 200 IU/day for the first three days and then 150 IU/day; after Day 6, a GnRH antagonist, Orgalutran (Organon), was administered daily by S.C. injection (0.25 mg/d) until the administration of recombinant human chorionic gonadotropin (rhCG) (Ovidrel 250 mg/0.5 ml, Serono). When three or more follicles reached 17 mm, oocyte maturation was stimulated with rhCG, and on this day, all follicles and serum FSH, LH, and P4 levels were measured. Ultrasound-guided oocyte retrieval was performed after 35 h and, following denudation, the oocytes were categorized as metaphase II (MII), metaphase I (MI), or prophase I (PI) stage. After oocyte retrieval, the patients were split into two groups: NOR (four or more oocytes) or POR (three or fewer oocytes).

Whole blood samples were collected and aliquots of 350 µL from each sample were used for genomic DNA extraction using the Easy DNA kit according to manufacturer’s instructions (Invitrogen, UK). Concentration and purity were determined by spectrophotometry NanoDrop ND1000 (NanoDrop Technologies Inc, USA). DNA was used for analysis of polymorphisms in the LHβ, LHR, GDF9, FSHR, AMH, AMHR2, and BMP15 genes (Table 1).

**Table 1:** Studied gene polymorphisms

Gene	Chromosome	Polymorphism^a^	Molecular biology techniques	Primer^a^
**GDF9^b^**	5	c.398-39 C>G (intron)	Direct sequencing	
		p.Thr149Thr (c.447C>T)		Forward: 5' TTGACTTGACTGCCTGTTGTG 3'
		p.Glu186Glu (c.546G>A)		Reverse: 5’ AGCCTGAGCACTTGTGTCATT 3’
		p.Val216Met (c.646G>A)		

**FSHR^c^**	2	p.Asn680Ser	RFLP - restriction enzyme BsrI	Forward: 5' TTGTGGTCATCTGTGGCTGC 3'
				Reverse: 5' AAAGGCAAGGACTGAATTATCATT 3'
**LHβ^d^**	19	p.Trp8Arg	Direct sequencing	Forward: 5’ GAAGCAGTGTCCTTGTCCCA 3’
		p.Ile15Thr		Reverse: 5’ GAAGAGGAGGCCTGAGAGTT 3’ Reverse: 5’ GGAGGGAAGGTGGCATAGAG 3’
**LHCGR^e^**	2	18insLQ	Direct sequencing	Forward: 5’ CACTCAGAGGCCGTCCAAG 3’
**AMH^f^**	19	p.Ile49Ser (c.146T>G)	Taqman	Assay ID: C_25599842_10
**AMHR2^g^**	12	c.-482A>G	Taqman	Assay ID: C_1673084_10
**BMP15^h^**	X	c.-9C>G	Taqman	Assay ID: C_27504454_10

a C: cytosine, T: thymine, A: adenine, G: guanine

b Human growth differentiation factor 9

c Follicle-stimulating hormone receptor

dh Bone morphogenetic protein 15

Polymerase chain reaction (PCR) was performed to amplify the regions of interest in the LHβ, LHR, FSHR and GDF9 genes (Table 1). Forward and reverse primers delimited the region where the polymorphisms were located, as described in Table 1. Amplification of DNA at a concentration of 200 ng was performed in a thermocycler (Veriti^®^ 96-Well Thermal Cycler, Applied Biosystems, USA) with reagent Invitrogen (UK). The following annealing temperatures were applied: -63ºC, LHR; -64ºC, GDF9; -63ºC, FSHR; and LH, 60ºC. PCR products were stained with SYBR^®^ Gold (Life Technologies, USA) and verified by electrophoresis on 1.5% agarose gel, and the DNA fragments obtained had the following sizes: LHβ - 662 base pairs, LHR - 350 pairs bases, GDF9 - 491 base pairs and FSHR - 520 base pairs.

The PCR products (detection of polymorphisms of LHβ, LHR and GDF9 - direct sequencing**)** were purified using the purification protocol with PEG8000 and 2.5% NaCl and subjected to direct sequencing by the Sanger method on an automated sequencer ABI 3500 Genetic Analyzer (Applied Biosystems, USA). Assays were performed at the Unit of Molecular and Protein Analysis of Hospital de Clinicas de Porto Alegre (UAMP/HCPA). Primers for sequencing were used at a concentration of 4 pmol/µL. The results were compared with NCBI reference sequences LHβ - NM_000894.2, LHR - NM_000233.3, and GDF9 - NM_005260.3 (Table 1).

The product of the PCR for the FSHR gene underwent RFLP using restriction enzyme BSRI for four hours at 65 º C. After digestion, the samples were stained with SYBR ^®^ Gold (Life Technologies, USA) and the results obtained were determined by electrophoresis on 2.5% agarose gel: NN (680Asn/Asn) fragments of 520 base pairs; SS (680Ser/Ser) fragments of 413 and 107 base pairs; and NS (680Asn/Ser) fragments of 520, 413 and 107 base pairs. The results were compared with the NCBI reference sequence NM_000145.3 (Table 1).

The polymorphisms in the AMH, AMHR2, and BMP15 genes were determined by TaqMan Allelic Discrimination - Real-Time PCR from DNA at a concentration of 10-20 ng. The tests shown in Table 1 (Applied Biosystems, USA) were performed on a StepOne ™ Real-Time PCR System (Applied Biosystems, USA) at the Unit of Analysis Molecular and Proteins Hospital de Clinicas de Porto Alegre (UAMP/HCPA).

Observed numbers for each genotype were compared against expected values to test whether the samples were in Hardy-Weinberg equilibrium using the Chi-Squared test with one degree of freedom. Data were tested for normality using the Kolmogorov-Smirnov test, and data with a normal distribution, as determined by the t-test, were used to compare means. The Kruskal-Wallis test was used for nonparametric data. Qualitative variables were analyzed with the Chi-squared test. Statistical analyses to determine the association between the polymorphisms were performed using the Chi-squared test and the Monte Carlo method for multiple simulations assuming that the null hypothesis was correct with fixed marginal values. Marginal values were determined from experimental data. This method determines an empirical *P* value for the observed data. Moreover, we utilized advanced statistical analysis to evaluate the effect of polymorphisms using logistic regression to predict the effect on POR. Statistical significance was set at *p*<0.05. Statistical tests were performed on the Statistical Package for the Social Sciences 23 (SPSS Inc., Chicago, IL).

## RESULTS

The clinical characteristics of patients undergoing COH for IVF are presented in Table 2. Mean age was similar (34.6 NOR versus 35.5 POR, *p*=0.271), and the women with NOR were heavier than the individuals with POR (65.9 NOR versus 56.4 POR, *p*=0.001).

**Table 2:** Clinical characteristics of patients submitted to controlled ovarian hyperstimulation for *in vitro* fertilization (mean±SD)

Parameter	Normal ovarian response n = 52	Poor ovarian response n = 14	*p* value^a^
**Age (years)**	34.6±3.3	35.5±3.7	0.271
**Infertility (years)**	3.9±2.68	5.3±4.7	0.208
**Menstrual cycles (days)**	29.1±3.98	28.3±2.25	0.504
**Ethnicity**			0.341^b^
**-Caucasian**	25	4	
**-African-American**	1	0	
**-Latin American**	26	10	
**Pregnancy (n)**	0.08±0.33	0.21±0.57	0.093
**BMIc (kg/m^2^)**	24.6±3.18	22.0±3.10	0.005
**Weight (kg) **	65.9±9.38	56.4±8.13	0.001
**Height (m)**	1.6±0.06	1.5±0.06	0.117

a T-test

b Chi-squared test

c BMI: Body mass index

Ovarian and hormonal characteristics of patients submitted to COH for IVF are presented in Table 3. Antral follicle count (12.3 NOR versus 4.2 POR, *p*<0.001), number of follicles greater than 17 mm at rhCG day (7.1 NOR versus 2.0 POR, *p*<0.001), MII oocyte count (9.8 NOR versus 1.7 POR, *p*<0.001), and AMH (2.0 NOR versus 0.6 POR, *p*=0.017) were lower in the POR group.

**Table 3:** Ovarian and hormonal characteristics of patients submitted to controlled ovarian hyperstimulation for *in vitro* fertilization (mean±SD)

Parameter	Normal ovarian response n = 52	Poor ovarian response n = 14	*p* value^a^
**Antral follicle count (n)**	12.3±4.6	4.2±1.5	<0.001
**Induction length (days)**	9.7±1.6	8.7±2.2	0.005
**Gonadotropin administration (RFSH) (UI)**	1586±281	1573±405	0.270
**Endometrial thickness (mm)**	9.9±1.8	8.9±2.1	0.095
**Follicles > 17 mm on rhCG Day (n)**	7.1±3.3	2.0±0.6	<0.001
**Follicles between 14-16 mm on rhCG day (n)**	3.3±2.0	1.0±0.8	<0.001
**Follicles between 12-14 mm on rhCG day (n)**	2.5±1.7	0.4±0.8	<0.001
**Total oocytes (n)**	12.4±7.4	2.1±0.9	<0.001
**MII oocytes (n)**	9.8±6.2	1.7±0.8	<0.001
**FSH Day 3 of menstrual cycle**	5.0±3.5	6.9±6.0	0.185
**E2 Day 3 of menstrual cycle**	56.4±41.2	59.0±58.3	0.850
**LH Day 3 of menstrual cycle**	4.5±3.3	4.2±1.5	0.721
**AMH**	2.0±2.4	0.6±0.5	0.017
**LH day rhCG**	1.6±1.9	1.4±1.1	0.071
**Progesterone day rhCG**	0.9±1.1	0.7±1.3	0.717
**E2 day rhCG**	1370.8±778.2	568.3±306.0	0.001
**Prolactin(ng/ml)**	21.3±17.6	18.2±19.2	0.694

a T-test

b Chi-squared test

c BMI: Body mass index

The test for Hardy-Weinberg equilibrium was performed via the Chi-squared test, and the following results were found: *p*=0.51 for GFD-9 398; *p*=0.33 for GFD-9 C447T; *p*=0.74 for GFD-9 546; *p*=0.51 for FSHR (Asn680Ser); *p*=0.60 for LH (Trp8Arg); *p*=0.60 for LH (Ile15Thr); *p*=0.99 for LHR (18isnLQ); *p*=0.16 for AMH (Ile49Ser); *p*=0.91 for AMHR2 (482A>G); and *p*=0.98 for BMP15 (9C>G) polymorphisms.

The genotype and allele frequencies of polymorphisms in the NOR and POR groups are demonstrated in Table 4. Two polymorphisms of GDF9 were associated with follicular response after COH. One was associated with POR: the presence of a mutant polymorphism in heterozygosis and homozygosis of the GDF9 398-39 (C to G) (23% NOR versus 68% POR; OR 4.01, IC 1.52-10.6, *p*=0.005); and the presence of normal homozygosis of GDF9 (C447T) were associated with protective response (19.2% NOR versus 50% POR; OR 0.34, IC 0.14-0.84, *p*=0.019). There was no association between other studied polymorphisms and POR.

**Table 4:** Genotype and allele frequencies of polymorphisms in women submitted controlled ovarian hyperstimulation for IVF with normal ovarian response (NOR) and poor ovarian response (POR)

Polymorphism	Population studied	N	Genotypes^b^			*p* value^a^	Alleles^b^		OR (95% CI)	*p* value
			n (%)	n (%)	n (%)		n (%)	n (%)		
** GDF9** **(398-39; C to G)**	NOR POR	52 13^d^	**CC** 40 (76.9%) 4(30.8%)	**CG** 10 (19.2%) 8(61.5%)	**GG** 2 (3.8%) 1(7.7%)	.006	**C** 90(86.5%) 16(61.5%)	**G** 14 (13.5%) 10 (38.5%)	4.01(1.52-10.60)	0.005
** GDF9** **(C447T)**	NOR POR	52 14	**CC** 10(19.2%) 7 (50.0%)	**CT** 24(46.2%) 5 (35.7%)	**TT** 18(34.6%) 2 (14.3%)	.049	**C** 44(42.3%) 19(67.9%)	**T** 60(57.7%) 9(32.1%)	0.34 (0.14-0.84)	0.019
** GDF9** **(G546A)**	NOR PORW	52 14	**GG** 37(69.2%) 11(78.6%)	**GA** 15(28.8%) 2 (14.3%)	AA 1(1.9%) 1(7.1%)	.362	**G** 89(85.6%) 24(85.7%)	**A** 17(16.4%) 4(14.3%)	0.87 (0.26-2.83)	0.821
** GDF9** **(G646A)**	NOR POR	52 14	**GG** 52(100) 14(100)	**AG** - -	**AA** - -	.c	**G** - -	**A** - -		.c
** FSHR** **(Asn680Ser)**	NOR POR	52 14	**NN** 18(34.6%) 2(14.3%)	**NS** 25(48.1%) 10(71.4%)	**SS** 9(17.3%) 2(14.3%)	.259	**N** 61(58.7%) 14(50.0%)	**S** 43(41.3%) 14(50.0%)	1.41(0.61-3.27)	0.410
** LH** **(Trp8Arg)**	NOR POR	52 14	**TT** 45(86.5%) 13(92.9%)	**TC** 7(13.5%) 1(7.1%)	**CC** 0(0) 0(0)	.520	**T** 97(93.3%) 27(96.4%)	**C** 7(6.7%) 1(3.6%)	0.51(0.06-4.35)	0.541
** LH** **(Ile15Thr)**	NOR POR	52 14	**TT** 45(86.5%) 13(92.9%)	**TC** 7(13.5%) 1(7.1%)	**CC** 0(0) 0(0)	.520	**T** 97(93.3%) 27(96.4%)	**C** 7(6.7%) 1(3.6%)	0.51(0.06-4.35)	0.541
** LHR** **(18isnLQ)**	NOR POR	52 14	**NN** 31(61.5%) 9(64.5%)	**NV** 18(34.6%) 4(28.6%)	**VV** 2(3.8%) 1(7.1%)	.903	**N** 80(78.4%) 22(78.5%)	**V** 22(21.6%) 5(21.5%)	0.82(0.28-2.43)	0.729
** AMH** **(Ile49Ser)**	NOR POR	52 14	**TT** 30(57.7%) 5(35.7%)	**TG** 21(40.4%) 8(57.1%)	**GG** 1(1.9%) 1(7.1%)	.206	**T** 81(77.9%) 18(64.3%)	**G** 23(22.1%) 10(35.7%)	1.95(0.79-4.81)	0.144
** AMH-R2** **(A to G)**	NOR POR	52 14	**AA** 41(78.8%) 9(64.3%)	**AG** 11(21.1%) 4(28.6%)	**GG** 0(0) 1(7.1%)	.117	**A** 93(89.4%) 22(78.5%)	**G** 11(10.6%) 6(21.5%)	2.30(0.76-6.91)	0.136
** BMP15** **(9C>G)**	NOR POR	52 14	**CC** 33(63.5%) 8(57.1%)	**CG** 16(30.8%) 6(42.9%)	**GG** 3(5.8%) 0(0)	.506	**C** 82(78.9%) 22(78.5%)	**G** 22(21.1%) 6(21.5%)	1.01(0.36-2.81)	0.975

a Monte Carlo test

b C: cytosine, T: thymine, A: adenine, G: guanine

c No statistics are computed because GDF9 G646A is a constant

d There was one less case in this group due to failure of DNA sequencing.

When we evaluated our data with logistic regression to investigate the influence of polymorphisms, only the GDF9 398 polymorphism was associated with POR after controlling for bias from other polymorphisms (Table 5). The logistic regression with other parameters of poor responders showed that AMH, gonadotropin, and the GDF9 C447 polymorphism were associated with POR.

**Table 5:** Logistic regression - influence of polymorphisms on ovarian response after controlled ovarian hyperstimulation for IVF

Unstandardized Coefficients B	Standardized Coefficients	Beta	95% Confidence Interval for B
Constant	3.087	0.393	21.916
GDF9 (398-39; C to G)	-1.361	0.039	0.257 (0.070-0.935)
GDF9 (C447T)	0.841	0.113	2.318 (0.820-6.555)
GDF9 (G546A)	0.675	0.296	1.964 (0.554-6.968)
FSHR (Asn680Ser)	-0.937	0.077	0.392 (0.139-1.107)
LH (Trp8Arg/Ile15Thr)	0.263	0.678	1.301 (0.376-4.498)
LHR (18isnLQ)	0.344	0.631	1.410 (0.346-5.730)
AMH (Ile49Ser)	-0.371	0.371	0.690 (0.306-1.555)
AMH R2 (A to G)	0.110	0.810	1.116 (0.457-2.723)
BMP15*(9C>G)	0.275	0.675	1.316 (0.364-4.759)

* Bone morphogenetic protein 15

## DISCUSSION

The present study revealed that GDF9 polymorphisms were associated with follicular response after COH for IVF. These polymorphisms can have a negative impact, i.e., the presence the allele mutant genotype in heterozygosis or homozygosis of GDF9 398-39 (C to G) was found in 69% of the individuals in the POR group and in only 23% of the women in the NOR group (OR 4.01). They may also have a protective response, i.e., the presence of normal homozygosis of GDF9 (C447T) was associated with POR (19.2% NOR versus 50% POR).

Multivariate analysis showed the possible influence of polymorphisms of genes regulating follicular growth and oocyte development (FSHR, LH, LHR, AMH, AMH2, BMP15 and GDF9). Response to administration of gonadotropins during ovarian hyperstimulation for IVF control showed that only the GDF9 398-39 (C to G) polymorphism was associated with POR, suggesting that this polymorphism plays an important role in the status of ovarian reserve and function.

We did not find correlations between the other studied polymorphisms, including GDF9 (G546A and G646A), FSHR (Asn680Ser), LH (Trp8Arg and Ile15Thr), LHR (18isnLQ), AMH (Ile49Ser), AMHR2 (482A>G), or BMP15 (9C>G), and POR. Unlike our study, other authors described associations between the FSHR (Livshyts *et al*., 2009; Sheikhha *et al*., 2011) and LHCGR polymorphisms (Skiadas *et al.,* 2012) and POR. A possible cause for this difference is the variety of ethnic groups within the populations included in each study.

GDF9 plays a pivotal role during early folliculogenesis, and deletion of GDF9 in mice causes follicular arrest at the primary stage and infertility (Dong *et al*., 1996). GDF9 also stimulates granulosa cell proliferation (Vitt *et al*., 2000), cumulus cell expansion (Yan *et al*., 2001), follicular apoptosis inhibition (Orisaka *et al*., 2006), and enhancement of oocyte and embryo development (Hussein *et al*., 2006; Yeo *et al*., 2008). *In vitro* studies using recombinant GDF9 protein have clarified the biological roles and the importance of GDF9 in follicular growth and development in all stages of folliculogenesis. In the preantral stage, GDF9 effectively stimulated the growth of *in vitro* cultured preantral follicles (Hayashi *et al*., 1999). GDF9 also promotes early preantral follicular growth in human ovaries (Hreinsson *et al*., 2002). In the transition to the antral stage, it appears that GDF9 promotes follicular survival by suppressing granulosa cell apoptosis and follicular atresia (Orisaka *et al*., 2006). This may be achieved in part by GDF9 stimulation of FSHR expression, since adequate FSRH levels in granulosa cells are essential for FSH-dependent antral follicle growth.

GDF9 also plays an important role during the final stages of follicular growth prior to ovulation. Prior to LH surge, cumulus cells require GDF9 to support metabolic cascades such as glycolysis and sterol biosynthesis (Sugiura *et al*., 2005). GDF9 also regulates diverse processes and gene expression during the preovulatory stage (Elvin *et al*., 2000) and enhances cumulus cell expansion in the presence of FSH (Elvin *et al*., 1999), but not without FSH (Dragovic *et al*., 2005). Although animal models, *in vitro* studies, and humans studies have revealed the role of GDF9 in regulating follicular development, little is known about it in human ovarian function, since studies have demonstrated a strong correlation between GDF9 polymorphism in women with DOR and poor ovarian response followed by poor IVF outcomes, indicating that GDF9 plays an important role in determining ovarian reserve status and function (Wang *et a*l., 2010b; 2013; Sanfins *et al*., 2018; Bilibio *et al*., 2020). In this study, we saw that the number of antral follicles, total follicles, oocytes retrieved, AMH, and estradiol day rhCG decreased in poor responders, suggesting that the GDF9 polymorphism affected oocyte development in all stages of folliculogenesis.

This study found a valuable association of polymorphisms of GDF9398 and ovarian response in patients undergoing ovarian stimulation for IVF in a relatively young and ethnically heterogeneous population. However, the total number of patients participating in this study must be considered. This is an element of great importance, since despite the number of included patients, we found significant results in an ethnically heterogeneous population. Our findings suggest that this polymorphism may be a predictor of ovarian response in other populations.

## CONCLUSION

The GDF9 polymorphism (398-39; C to G and C447T) exerts an important influence on oocyte development. GDF-9 seems to influence all stages of folliculogenesis by decreasing follicular recruitment, decreasing ovarian response, or decreasing the number of MII specimens after ovarian hyperstimulation for IVF. Functional studies are required to understand the real function and a possible correction and treatment to improve the number of oocytes retrieved in patients with this polymorphism.

## References

[r1] Aaltonen J, Laitinen MP, Vuojolainen K, Jaatinen R, Horelli-Kuitunen N, Seppä L, Louhio H, Tuuri T, Sjöberg J, Bützow R, Hovata O, Dale L, Ritvos O (1999). Human growth differentiation factor 9 (GDF-9) and its novel homolog GDF-9B are expressed in oocytes during early folliculogenesis. J Clin Endocrinol Metab.

[r2] Abir R, Ben-Haroush A, Melamed N, Felz C, Krissi H, Fisch B (2008). Expression of bone morphogenetic proteins 4 and 7 and their receptors IA, IB, and II in human ovaries from fetuses and adults. Fertil Steril.

[r3] Alviggi C, Clarizia R, Pettersson K, Mollo A, Humaidan P, Strina I, Coppola M, Ranieri A, D’Uva M, De Placido G (2009). Suboptimal response to GnRHa long protocol is associated with a common LH polymorphism. Reprod Biomed Online.

[r4] Bilibio JP, Meireles AJC, Conto E, Lorenzzoni PL, Nascimento FCD, Cunha-Filho JSD (2020). GDF9 polymorphisms: influence on ovarian response in women undergoing controlled ovarian hyperstimulation. JBRA Assist Reprod.

[r5] Chang HM, Qiao J, Leung PC (2016). Oocyte-somatic cell interactions in the human ovary-novel role of bone morphogenetic proteins and growth differentiation factors. Hum Reprod Update.

[r6] de Boer EJ, den Tonkelaar I, te Velde ER, Burger CW, Klip H, van Leeuwen FE, OMEGA-project group (2002). A low number of retrieved oocytes at in vitro fertilization treatment is predictive of early menopause. Fertil Steril.

[r7] Di Pasquale E, Beck-Peccoz P, Persani L (2004). Hypergonadotropic ovarian failure associated with an inherited mutation of human bone morphogenetic protein-15 (BMP15) gene. Am J Hum Genet.

[r8] Dong J, Albertini DF, Nishimori K, Kumar TR, Lu N, Matzuk MM (1996). Growth differentiation factor-9 is required during early ovarian folliculogenesis. Nature.

[r9] Dragovic RA, Ritter LJ, Schulz SJ, Amato F, Armstrong DT, Gilchrist RB (2005). Role of oocyte-secreted growth differentiation factor 9 in the regulation of mouse cumulus expansion. Endocrinology.

[r10] Elvin JA, Yan C, Matzuk MM (2000). Growth differentiation factor-9 stimulates progesterone synthesis in granulosa cells via a prostaglandin E2/EP2 receptor pathway. Proc Natl Acad Sci U S A.

[r11] Elvin JA, Yan C, Wang P, Nishimori K, Matzuk MM (1999). Molecular characterization of the follicle defects in the growth differentiation factor 9-deficient ovary. Mol Endocrinol.

[r12] Ferraretti AP, La Marca A, Fauser BC, Tarlatzis B, Nargund G, Gianaroli L, ESHRE working group on Poor Ovarian Response Definition (2011). ESHRE consensus on the definition of ‘poor response’ to ovarian stimulation for in vitro fertilization: the Bologna criteria. Hum Reprod.

[r13] Ferrarini E, Russo L, Fruzzetti F, Agretti P, De Marco G, Dimida A, Gianetti E, Simoncini T, Simi P, Baldinotti F, Benelli E, Pucci E, Pinchera A, Vitti P, Tonacchera M (2013). Clinical characteristics and genetic analysis in women with premature ovarian insufficiency. Maturitas.

[r14] Goswami D, Conway GS (2005). Premature ovarian failure. Hum Reprod Update.

[r15] Greene AD, Patounakis G, Segars JH (2014). Genetic associations with diminished ovarian reserve: a systematic review of the literature. J Assist Reprod Genet.

[r16] Hanevik HI, Hilmarsen HT, Skjelbred CF, Tanbo T, Kahn JA (2011). A single nucleotide polymorphism in BMP15 is associated with high response to ovarian stimulation. Reprod Biomed Online.

[r17] Hayashi M, McGee EA, Min G, Klein C, Rose UM, van Duin M, Hsueh AJ (1999). Recombinant growth differentiation factor-9 (GDF-9) enhances growth and differentiation of cultured early ovarian follicles. Endocrinology.

[r18] Hreinsson JG, Scott JE, Rasmussen C, Swahn ML, Hsueh AJ, Hovatta O (2002). Growth differentiation factor-9 promotes the growth, development, and survival of human ovarian follicles in organ culture. J Clin Endocrinol Metab..

[r19] Hussein TS, Thompson JG, Gilchrist RB (2006). Oocyte-secreted factors enhance oocyte developmental competence. Dev Biol..

[r20] Lawson R, El-Toukhy T, Kassab A, Taylor A, Braude P, Parsons J, Seed P (2003). Poor response to ovulation induction is a stronger predictor of early menopause than elevated basal FSH: a life table analysis. Hum Reprod.

[r21] Levi AJ, Raynault MF, Bergh PA, Drews MR, Miller BT, Scott Jr RT (2001). Reproductive outcome in patients with diminished ovarian reserve. Fertil Steril.

[r22] Livshyts G, Podlesnaja S, Kravchenko S, Sudoma I, Livshits L (2009). A distribution of two SNPs in exon 10 of the FSHR gene among the women with a diminished ovarian reserve in Ukraine. J Assist Reprod Genet.

[r23] Morón FJ, de Castro F, Royo JL, Montoro L, Mira E, Sáez ME, Real LM, González A, Mañes S, Ruiz A (2006). Bone morphogenetic protein 15 (BMP15) alleles predict over-response to recombinant follicle stimulation hormone and iatrogenic ovarian hyperstimulation syndrome (OHSS). Pharmacogenet Genomics.

[r24] Orisaka M, Orisaka S, Jiang JY, Craig J, Wang Y, Kotsuji F, Tsang BK (2006). Growth differentiation factor 9 is antiapoptotic during follicular development from preantral to early antral stage. Mol Endocrinol.

[r25] Pabalan N, Trevisan CM, Peluso C, Jarjanazi H, Christofolini DM, Barbosa CP, Bianco B (2014). Evaluating influence of the genotypes in the follicle-stimulating hormone receptor (FSHR) Ser680Asn (rs6166) polymorphism on poor and hyper-responders to ovarian stimulation: a meta-analysis. J Ovarian Res.

[r26] Sanfins A, Rodrigues P, Albertini DF (2018). GDF-9 and BMP-15 direct the follicle symphony. J Assist Reprod Genet.

[r27] Sheikhha MH, Eftekhar M, Kalantar SM (2011). Investigating the association between polymorphism of follicle-stimulating hormone receptor gene and ovarian response in controlled ovarian hyperstimulation. J Hum Reprod Sci.

[r28] Shimizu T, Miyahayashi Y, Yokoo M, Hoshino Y, Sasada H, Sato E (2004). Molecular cloning of porcine growth differentiation factor 9 (GDF-9) cDNA and its role in early folliculogenesis: direct ovarian injection of GDF-9 gene fragments promotes early folliculogenesis. Reproduction.

[r29] Skiadas CC, Duan S, Correll M, Rubio R, Karaca N, Ginsburg ES, Quackenbush J, Racowsky C (2012). Ovarian reserve status in young women is associated with altered gene expression in membrana granulosa cells. Mol Hum Reprod.

[r30] Sugiura K, Pendola FL, Eppig JJ (2005). Oocyte control of metabolic cooperativity between oocytes and companion granulosa cells: energy metabolism. Dev Biol..

[r31] Vitt UA, Hayashi M, Klein C, Hsueh AJ (2000). Growth differentiation factor-9 stimulates proliferation but suppresses the follicle-stimulating hormone-induced differentiation of cultured granulosa cells from small antral and preovulatory rat follicles. Biol Reprod.

[r32] Wang JG, Douglas NC, Nakhuda GS, Choi JM, Park SJ, Thornton MH, Guarnaccia MM, Sauer MV (2010a). The association between anti-Müllerian hormone and IVF pregnancy outcomes is influenced by age. Reprod Biomed Online.

[r33] Wang TT, Wu YT, Dong MY, Sheng JZ, Leung PC, Huang HF (2010b). G546A polymorphism of growth differentiation factor-9 contributes to the poor outcome of ovarian stimulation in women with diminished ovarian reserve. Fertil Steril.

[r34] Wang TT, Ke ZH, Song Y, Chen LT, Chen XJ, Feng C, Zhang D, Zhang RJ, Wu YT, Zhang Y, Sheng JZ, Huang HF (2013). Identification of a mutation in GDF9 as a novel cause of diminished ovarian reserve in young women. Hum Reprod.

[r35] Yan C, Wang P, DeMayo J, DeMayo FJ, Elvin JA, Carino C, Prasad SV, Skinner SS, Dunbar BS, Dube JL, Celeste AJ, Matzuk MM (2001). Synergistic roles of bone morphogenetic protein 15 and growth differentiation factor 9 in ovarian function. Mol Endocrinol.

[r36] Yeo CX, Gilchrist RB, Thompson JG, Lane M (2008). Exogenous growth differentiation factor 9 in oocyte maturation media enhances subsequent embryo development and fetal viability in mice. Hum Reprod.

